# Factors affecting HPV vaccine acceptance in west Austria: Do we need to revise the current immunization scheme?

**DOI:** 10.1016/j.pvr.2016.10.001

**Published:** 2016-10-19

**Authors:** Wegene Borena, Anita Luckner-Hornischer, Franz Katzgraber, Dorothee Holm-von Laer

**Affiliations:** a Division of Virology, Medical University of Innsbruck, Austria; b Local Public Health Department, Austria

**Keywords:** HPV vaccine, Educational status, Age of vaccination, Gender-neutral, School-based

## Abstract

**Background:**

Austria introduced a school-based gender-neutral human papillomavirus (HPV) immunization program in February 2014. In order to assure high coverage, factors influencing acceptance of the vaccine need to be identified. In this study we aim to assess parents’ attitude and related socio-demographic factors in relation to the newly implemented gender-neutral, school-based HPV Immunization program.

**Methods:**

Parents of 4th grade school children in 20 randomly selected primary schools were asked to fill out questionnaires on socio-demographic factors and on the level of information and attitude towards HPV infection and HPV vaccine.

**Results:**

A total of 439 parents with 449 vaccine eligible children participated in the study. Fifty nine percent of vaccine eligible girls and 51.8% of eligible boys received the first dose of the vaccine. Fear of side effects and child being too young for the vaccine were the most commonly cited reasons by parents electing not to let child receive the vaccine. Children who had received other school-based vaccines have more than fifteen times higher probability of receiving HPV vaccine. To have received HPV-related information from physicians positively influenced vaccine acceptance (OR (95% CI)=1.60 (1.06–2.43)). Higher paternal (fathers’) educational status significantly increased the chances of a male child to be HPV vaccinated (OR (95% CI)=2.45 (1.29–4.78)).

**Conclusion:**

Despite the efforts to provide HPV vaccine free-of-costs and as a school-based program, the study found that a significant proportion of vaccine eligible children failed to receive the vaccine. Involvement front line physicians and men with higher educational status may be utilised by public health policy makers in the effort to increase awareness. For a better acceptability of the vaccine, there is a need to consider lifting the age of “eligibility” for the school-based vaccination program.

## Highlights

• Paternal (Fathers') educational status positively influences HPV vaccine acceptance, particularly vaccination of a male child.
• Involving general practitioners and paediatricians in providing the vaccine may increase the acceptability.
• Lifting the vaccine eligibility age may need to be considered in the effort to raise HPV vaccine acceptability.

## 1 Background

Human papillomavirus (HPV) infection is a common sexually transmitted infection peaking in prevalence in adolescence [1]. Most of these infections are transient and only a certain proportion persists to lead to high grade lesions and eventually to HPV associated cancers [2], [3], [4], [5], [6].

For the prevention of cervical and other genital neoplasia as well as genital warts, three vaccines – Gardasil® *(quadrivalent vaccine against HPV types 6, 11, 16 and 18),* Gardasil 9® *(nonavalent vaccine against HPV types 6, 11, 16, 18, 31, 33, 45, 52 and 58)* and Cervarix® *(bivalent vaccine against HPV types 16 and 18)* – have been developed and approved for use [7], [8], [9]. Even if the vaccines had been recommended to young men and women in Austria, free-of-charge immunization program was started only in 2014 [10]. Starting from the autumn of 2014, fourth grade primary school girls and boys receive two doses of Gardasil® (0–6 months) as part of school-based immunization program. This makes Austria – next to Australia, USA and some parts of Canada – the first country to incorporate men/boys in the routine HPV immunization program [11].

Estimations from mathematical modelling studies reveal that a gender-neutral immunization scheme may result in a substantial and more rapid reduction in the associated disease burden [12]. This goal of rapid reduction in disease burden can only be reached depending on the proportion of target population who receive the vaccine. Seen generally, one of the most important factors affecting the immunization coverage is the acceptance of the vaccine in the community. Previous studies in other countries have shown that parents' level of information and attitude towards HPV vaccine play decisive role in the acceptance of the vaccine [13], [14], [15], [16], [17].

In Austria - due to the novelty of the program - there are no data on parental attitudes and acceptance towards HPV vaccine.

With this study, we aim to assess parental factors associated with decision to let child receive the newly introduced gender-neutral, free-of-charge, school-based HPV immunization. The results of the survey will be of high value in evaluating and tackling the possible barriers to successful coverage of the vaccine at this very early stage of the program.

## 2 Methods

### 2.1 Study design

This is a cross sectional survey on the knowledge, attitude and practice of parents of primary school children in west Austria on the newly implemented school-based HPV vaccination program.

### 2.2 Study population

#### 2.2.1 Study participants

The study participants were parents of a total of 671 fourth grade school children from 20 primary schools in Tyrol – a region in west Austria. The schools were selected randomly out of a total of 383 primary schools in the region [18]. The study was conducted in April 2015.

#### 2.2.2 Data collection

After obtaining ethical clearance from the Ethic Committee of the Medical University of Innsbruck and the regional school inspector, questionnaires were distributed to the parents via the respective schools. The information obtained from the questionnaires included socio-demographic data, awareness on HPV Infection, knowledge and attitude towards school-based immunizations particularly on HPV vaccine. School children brought filled out and sealed questionnaires back to the schools which subsequently were sent to the study center (local public health department of Tyrol) via a prepaid post system.

#### 2.2.3 Data analysis

Descriptive analyses of data were conducted. Logistic regression model was used to compute odds ratios with corresponding 95% confidence intervals for HPV vaccine acceptance across several socio demographic variables and other factors associated with awareness on HPV infection. P-values <0.05 were considered significant.

Statistical analyses were performed in SPSS (Version 20.0. Armonk, NY: IBM Corp.).

## 3 Results

A total of 439 study participants with 449 vaccine eligible boys and girls returned filled out questionnaires making a total response rate of 67%. Table 1 presents baseline socio-demographic characteristics of the study participants. Mean age of the study participants was 40.8 (SD=5.7). Majority of the respondents were females/mothers. Majority are married or live in partnership. Respondents and the respective partners have comparable educational as well as employment status.

**Table 1:** Baseline characteristics of survey respondents (n=439).

**Age, years**	
Mean (SD)	40.8 (5.7)
**Respondent, n (%)**	
Mother	395 (90%)
Father	42 (9.6%)
**Number of children**	
Median (IQR)	2 (2–3)
**Married/Partnership, n (%)**	380 (85.4)
**Religion, n (%)**	
R. Catholic	331 (74.4)
Muslim	47(10.6)
**Education, n (%)**	
Basic schooling	182 (41.5)
Higher education	257 (58.5)
**Partner's education, n (%)**	
Basic schooling	168 (43.8)
Higher education	216 (56.2)
**Employment status, n (%)**	340 (76.4)
**Partner's employment status, n (%)**	336 (82.2)
**Migration history, n (%)**	47 (10.6)

SD=standard deviation, IQR=interquartal range.

Table 2 shows data on awareness of HPV infection as well as on HPV vaccine acceptance. Majority of the study participants report to have heard about HPV and answered correctly, that HPV can cause cancer in both men and women. Only a quarter believe that HPV causes cancer only in women. One third of the study participants reported to have obtained information about HPV vaccine from their general practitioners or paediatricians whereas the rest received from the information-leaflets prepared by the local health government and sent out to the schools.

**Table 2:** Characteristics of survey participants across data relating to HPV infection and HPV vaccine (n=439).

**Heard about HPV, n (%)**	380 (85.4)
**HPV causes cancer, n (%)**	
Only in women	106 (23.8)
Only in men	0
Men and women	297 (66.7)
HPV causes no cancer	22 (4.9)
**HPV associated illness in the family**	66 (14.8)
**Source of HPV vaccine information, n (%)**	
Child's school	415 (93.3)
GP/paediatrician	145 (32.6)
**HPV Vaccine eligible children, n (%)**	
Vaccine eligible boys	195 (41.7)
Vaccine eligible girls	254 (57.9)
**HPV Vaccinated children, n (%)**	
Vaccinated boys	101(51.8)
Vaccinated girls	150 (59)
**Accept HPV vaccine later**^a^**, n (%)**	
Yes	48 (32.4)
No	29 (15.4)
Do not know	71 (37.8)
**Child vaccinated for other school-based vaccinations, n (%)**	379 (86.3)

HPV=human papillomavirus

a Among non-vaccinated, total number of respondents (n)=148.

Over 85% of the respondents reported that their children had received other school-based immunizations. Out of a total of 449 HPV vaccine eligible children 101 boys (51.8%) and 150 girls (59%) received the first dose of HPV the vaccine (Table 2).

Table 3 presents the odds ratios and 95% confidence intervals (OR (95% CI)) for factors affecting parents’ decisions to let child receive vaccine. Having child receive other school-based vaccines was associated with over a fifteen-fold higher probability of being vaccinated for HPV. Parents who report to have heard of HPV were significantly more likely to have child receive the vaccine. Moreover, having obtained information on HPV vaccine from physicians associated positively with the decision to accept the vaccine. The association was statistically significant for overall and female child vaccinations.

**Table 3:** Multivariate adjusted* factors affecting parents' decision to have child receive vaccine.

	**OR (95% CI)**		
**Factors**	**Overall**	**Girls (n=254)**	**Boys (n=195)**
***Respondents age (years)***			
≤40	1	1	1
>40	1.07 (0.73–1.58)	1.11 (0.67–1.86)	0.92 (0.52–1.62)
***Number of Children***			
One or two	1	1	1
Three or more	0.95 (0.78–1.16)	0.62 (0.37–1.05)	1.50 (0.82 −2.75)
***Marital status***			
Single/divorced	1	1	1
Married/partnership	0.89 (0.51–1.54)	0.99 (0.48–2.04)	0.69 (0.30–1.59)
***Educational status***			
Basic/vocation school	1	1	1
High school/university	1.25 (0.84–1.86)	1.37 (0.80–2.35)	1.20 (0.68–2.15)
***Educ. status (partner)***			
Basic/vocation school	1	1	**1**
High school/university	**1.75 (1.13–2.72)**†	1.31 (0.73–2.36)	**2.45 (1.29–4.78)**†
***Employment status***			
Non-employed	1	1	1
Employed/own business	1.21 (0.76–1.93)	1.62 (0.85–3.10)	0.90 (0.47–1.74)
***Employ. Status (partner)***			
Non-employed	1	1	1
Employed/own business	1.43 (0.45–4.53)	1.71 (0.24–12.41)	1.18 (0.29–4.87)
***Immigration status***			
Non-immigrant	1	1	1
Immigrant	0.57 (0.46–1.58)	0.83 (0.39–1.74)	0.91(0.31–2.62)
***Vaccinated for other school-based vaccines***			
No	1	**1**	**1**
yes	**15.8 (6.62–37.8)**†	**15.5 (5.26–44.9)**†	**18.5 (4.22–81.2)**†
***Heard of HPV***			
No	1	1	1
yes	**1.74 (1.**10**–2.79)**†	1.79(0.97–3.31)	1.45 (0.70–3.00)
***Respondent's estmation of HPV prevalence***			
HPV infection is rare	1	**1**	1
HPV infection is common	**1.80 (1.20–2.68**)†	**1.71 (1.00–2.90)**†	1.69 (0.93–3.07)
***HPV vaccine information from physician***			
No	1	1	1
yes	**1.60 (1.06–2.43)**†	**1.80 (1.05–3.10**)†	1.22 (0.65–2.29)

***Family history of HPV related diseases***			
No	**1**	1	1
Yes	**1.73(0.98–3.07)**	1.40 (0.70–2.81)	2.20 (0.87–5.59)

OR=odds ratio, CI=confidence interval, HPV=human papillomavirus

* Adjusted for age of respondents and number of children per family,

† = P>0.05.

Educational status of the male partner positively influenced HPV vaccine acceptance. This was particularly statistically significant for vaccination of boys whose fathers had reached levels of high school or above. On the contrary, neither respondents' (women's) educational status nor other socio-demographic factors like religion, marital, employment or immigration status showed significance on HPV vaccine acceptance.

The most commonly cited reasons for not having child receive HPV vaccine included, fear of debilitating or temporary side effects, child being too young for the vaccine, vaccine being too new and lack of adequate information on the vaccine (Table 4). Approximately ten percent of the study participants (one fifth of those who refused the vaccine) reported to be “generally against all vaccines”. About five percent of the parents who declined the vaccine gave the reason that their general practitioner or paediatrician was not convinced about the necessity of HPV vaccine. Most parents gave a combination of factors; however, child's age at vaccination was the most commonly cited single reason for opting out. Separate analysis of data for boys and girls showed no difference in the stated argumentations for declining the vaccine (data not shown).

**Table 4:** Main reasons for NOT having child receive HPV vaccine (n=185).

**Reasons, n (%)**	
Fear of debilitating/permanent side effects	56 (30.2)
Child too young for the vaccine	54 (29.2)
Fear of side effects although temporary	49 (26.5)
Not being adequately informed	42 (22.7)
Discouraging information about the vaccine from internet	40 (21.6)
Generally against all vaccines	37 (20)
Other reasons/Vaccine too new (majority)	33 (17.8)
The vaccine is just a publicity ploy by pharmaceutical company	31(16.8)
Child missed school at the day of vaccination	18 (9.7)
Vaccine unnecessary due to low disease risk	18 (9.7)
Vaccine not effective	15 (8.1)
Child is afraid of needles	14 (7.6)
Wait for vaccine which covers more HPV types	12 (6.5)
Child afraid of getting vaccinated at school	12 (6.5)
Child has allergy	11(5.9)
GP/Paediatrician does not believe it is necessary	10 (5.4)
Other reasons	20 (10.8)

## 4 Discussion

The current school-based HPV immunization program in Austria is designed to efficiently offset possible financial and logistic barriers that may interfere with vaccine acceptance. Despite these efforts, however, a significant proportion of the eligible target population, namely 50% of the boys and 40% of the girls, failed to receive the first dose of the HPV vaccine making the acceptance way below the expected vaccine coverage needed for a herd immunity effect [19]. Socio-demographic factors and factors associated with perception about HPV infection and vaccination were among the contributing factors.

Previous studies report statistically significant association between parental educational status and HPV vaccine acceptance. Majority of these reports indicated a paradoxical outcome of higher parental education having negative effect on HPV vaccination [13], [14], [15], [16]. A large register-based study in Norway based on about 85 000 study participants noted such an association both for maternal and paternal educational status, the degree of association being higher for that of maternal education [20]. These findings of inverse association are explained mainly by the fact that educated parents tend to undertake own internet research making them likely to end up in websites with unclear and confusing information on HPV or even websites which clearly condemn the vaccine [21], [22], [23]. One can also speculate that educated parents may be able to perceive HPV as “not-immediately-life-threatening” and may decide to postpone the vaccination to a later age, but still before first sexual contact.

Our result of paternal (male partners') educational status positively influencing HPV vaccine acceptance is in line with another population-based cohort study from Sweden [24], which showed fathers’ education at high school or university level to be significantly higher among HPV vaccinated individuals compared to non-vaccinated (relative risk (95% confidence interval) (RR (CI))=2.75 (2.69–2.81) and 4.31 (4.22–4.4)), respectively. This study found significant association not only for fathers’ but also for that of mothers' educational status. In our study, on the contrary, mothers' educational status, showed no statistically significant difference on HPV vaccine acceptance. Of note, the significant influence of paternal education for vaccination of boys but not girls is a unique finding to our study. Larger population based study may be needed to further clarify if the observed disparity between maternal and paternal educational status in our setting is a true difference or simply lack of a statistical power.

Further, our results demonstrated that awareness on HPV significantly impacted parents’ receptiveness to HPV vaccination. Although the proportion of parents who reported to have received information on the vaccine from their physicians was low, these parents were significantly more likely to let child receive the vaccine than those who had other sources of information. Encouraging physicians to make use of this encounter to provide information on HPV vaccine may be an important entry point in the effort to raise HPV vaccine coverage.

Vaccination of eligible children falling into the free-of-charge-immunization program in the study region is limited to schools (school-based) and to the local public health officers in the region [10]. This exclusion of general practitioners and paediatricians from providing the vaccine to the eligible group may be the reason why only a third of the participants got consulted by their physicians. It could be speculated that physicians may actively recommend the vaccine only if they also provide it. Studies from other regions with different immunization guidelines – where also paediatricians vaccinate the eligible age group – may be needed to assess the significance of this speculation.

Physician-patient encounter may also be used as a platform to deal with the commonly cited concerns of parents who decline the vaccine. In our study, “vaccine safety” was the most commonly stated reason for not having child receive HPV vaccine. Since other sources of information, like the web for example, report predominantly on speculated negative outcomes of vaccines in general and HPV vaccine in particular, front line physicians' (GPs and paediatricians) active and competent communication of the issue with their patients is of paramount importance.

Another major concern parents mentioned in the current study was “age of child at vaccination”. Almost 30% of parents who refused to let child receive the HPV vaccine stated that their nine- or ten-year-old child is too young for the vaccine. Since it is clear that HPV vaccine prevents sexually transmitted diseases, it may be speculated that parents find it incomprehensible to vaccinate a child for an effect needed several years later. In addition, an unpublished survey in the same study region among paediatricians and gynaecologists showed that about half of the physicians recommended vaccinating children at or beyond 12 years of age but definitely before sexual debut. Moreover, it is not well established if a 2-dose vaccination program provides a long-lasting protection as the 3-dose program, thereby not (yet) excluding the issue of a potential need for a booster immunization at the age where the vaccinees actually need the required effect [25]. The combination of these factors justifies a consideration to lift the age of vaccination by few years and yet before sexual debut. According to our findings, raising the age may highly likely increase the acceptance rate because only 15% of the parents clearly declined the vaccine even at a higher age and because only a minority are generally against all vaccines.

The chances of receiving HPV vaccine was shown to be more than fifteen times higher among eligible boys and girls who – according to the parents – had received other school-based vaccines. This may mean that barriers to HPV vaccine acceptance are not limited to the concerns associated with this particular vaccine but to a great extent also to the existing universal reservation towards all vaccines. Our study found a non-negligible proportion of the study participants to be generally against vaccines. This is in line with previous studies in Austria noting that immunization coverages had been on the decline leading to ongoing outbreaks in vaccine-preventable childhood diseases [26] Therefore, public health efforts fighting to tackle barriers to other childhood and school-based vaccine acceptance in general may also raise HPV vaccine coverage.

One limitation of our study may be the design of the questionnaire which was restricted to finding out the reasons for declining HPV vaccine but not for accepting it. Explicit factors stated by respondents for letting child receive vaccine may be important entry-points in programs initiated to raise awareness. Further limitation of the study may be the size of the study participants, which may have contributed to the lacking statistical significance in the associations between HPV vaccine acceptance and some factors which, in other studies, showed obvious significance. However, our study had enough power to demonstrate some significant and unique associations contributing valuable information to the existing pool of data in this area. Although the current study is limited to the western region of the country, the results motivate to undertake further surveys involving other regions of the country and compare factors influencing HPV vaccine acceptance.

In conclusion, this study presents the first data on the acceptance of the newly implemented gender-neutral HPV immunization program in Austria. Only half of the eligible population in this study received the vaccine. Most commonly cited reasons for vaccine rejection were safety concerns and age of child at vaccination. Our results established the importance of some socio-demographic and awareness related factors in significantly influencing HPV vaccine acceptance despite offsetting financial and logistic barriers through a free-of-charge school-based immunization policy. Front line physicians and educated men were found to be important stakeholders in HPV vaccine acceptability in the region. Educated men's involvement in the community as group or opinion leaders may be used as a vehicle to transport awareness on vaccines in general as well as on HPV vaccine in particular. Lifting the age of eligibility for the school-based HPV immunization program and involving first line physicians in providing the vaccine are key points worthy of consideration in the effort to increase the vaccine coverage.

## 5 Declarations

### 5.1 Ethics

This study was declared by the Ethical Committee of the Medical University of Innsbruck as a survey not requiring ethical approval (email communication is attached as a supplementary material).

### 5.2 Competing interests

The authors declare that they have no competing interests.
